# Prevalence of Rheumatic Diseases in a Tertiary Care Hospital of Karachi

**DOI:** 10.7759/cureus.2858

**Published:** 2018-06-22

**Authors:** Zainab Mohsin, Arifa A Asghar, Arisha Faiq, Ilma Khalid, Ibtehaj Ul-Haque, Sameen Rehman, Saffia I Ahmed, Syeda Tooba Basalat, Abeeha Aimen, Shiza Shafique, Ayesha Hanif, Muhammad Waqas Iqbal, Syed A Samad, Faiza Siddiqui, Ishaque Hameed, Marzia Safri

**Affiliations:** 1 Dow Medical College, Dow University of Health Sciences (DUHS), Karachi, PAK; 2 Dow Medical College, Dow University of Health Sciences (DUHS), Karachi , PAK; 3 Dow University of Health Sciences, Civil Hospital, Karachi, PAK; 4 Dow Medical College, Dow University of Health Sciences (DUHS), Karachi, Pakistan, Karachi, PAK

**Keywords:** rheumatic disease, osteoarthritis, non-specific low back pain, rheumatoid arthritis, tertiary care hospital, musculoskeletal disorders

## Abstract

Background:

Rheumatic diseases are referred to as conditions affecting joints, muscles, ligaments, tendons, and bones. According to a report by World Health Organization, rheumatic and musculoskeletal diseases were labeled as the second most reported cause of disability around the globe. The purpose of this study was to estimate the prevalence of rheumatic diseases in a tertiary care hospital of Karachi; additionally, associations with age groups, gender and comorbidities were obtained as well.

Methods:

A cross-sectional study was conducted at the Orthopedic Out Patient Department (OPD) of Dr. Ruth KM Pfau Civil Hospital, Karachi over a span of three months in 2018 (February till May). All 346 patients were follow-up diagnosed cases in the age range of 11-90 years, divided into groups of adolescents, young adults, adults, and older adults. The subjects were questioned about their symptoms, duration of illness, presence of comorbidities, genetic background and the therapy they are undergoing along with compliance. Simple statistical analysis of frequency was done, whereas chi-square test was applied to study associations with gender, age groups, and comorbidities.

Results:

During the study period, a total of 2000 patients visited the orthopedic OPD, 346 of which were diagnosed cases of rheumatic diseases, yielding a prevalence of 17.3%. The mean age of rheumatic patients who partook in the study was 46.15 ± 15.49 (Range: 12 – 84). Osteoarthritis was recorded as the most prevalent condition, followed by non-specific low back pain and rheumatoid arthritis. Osteoarthritis was statistically significant in young adults, adults, and older adults, while non-specific low back pain had significant associations with gender, young adults, and adults. Diabetes was significantly associated with osteoarthritis, non-specific low back pain, shoulder pain syndrome and psoriatic arthritis, while hypertension significantly co-existed with systemic lupus erthematosus.

Conclusion:

Rheumatic diseases constitute a major disease burden in almost all of the age groups, especially in young patients (18-40 years) within our setup.

## Introduction

The term ‘rheumatic diseases’ refers to roughly 200 conditions that affect joints, muscles, ligaments, tendons, and bones; with arthritis representing rheumatic conditions solely confined to joints. The most commonly known rheumatic diseases are osteoarthritis, rheumatoid arthritis and gouty arthritis. One third of the world population suffers from rheumatic diseases inflicting enormous financial price globally, with Europe bearing almost 200 billion euros per annum [1]. Being the prime cause of disability in developing countries, rheumatic diseases have the highest incapacitating rates in health-related quality of life (HRQoL) and daily functioning [1]. In the year 2005, 27 million individuals were suffering from osteoarthritis in the United States, while it emerged as the fourth major cause of hospital admissions in 2009 [2].

In reference to researches carried out in the recent years on disabled people, the World Health Organization has reported rheumatic and musculoskeletal diseases as the second most known cause of disability around the globe [3]. Although the exact epidemiologic statistics of all rheumatic diseases are not known in developing countries till date; studies showed that prevalence of rheumatoid arthritis was 0.55% in northern Pakistan and 0.142% in urban communities of Karachi. [4,5]. Rheumatic diseases result in extensive functional deterioration, with some rheumatic entities being more disabling than others when they occur in conjunction with comorbidities, while some are still under debate [6-8]. With little recent study on the prevalence of rheumatic diseases in young adults [9], our study focuses on the four major at-risk age groups for the development of such diseases. Since the chronic complications of these diseases pose a higher risk of morbidity and mortality, this study is directed to emphasize the need to devise a more productive protocol for their early detection and management.

This work, primarily, aimed to assess the prevalence of rheumatic diseases in the orthopedic outpatient department (OPD) of a tertiary care hospital in Karachi which largely caters to the under-privileged population from rural areas all over Pakistan. The secondary goal of this study was to learn about the frequency of various rheumatic diseases within different age groups and their association with gender and comorbidities.

## Materials and methods

Over a span of three months (February till May) in the year 2018, a cross-sectional study was conducted by a group of medical students after approval from the institutional review board of Dow University of Health Sciences. It aimed to assess the prevalence of different rheumatic diseases in a tertiary care hospital i.e. Dr. Ruth KM Pfau Civil Hospital, Karachi. A self-administered questionnaire was used to acquire study related information from the diagnosed patients visiting the Orthopedic OPD of the hospital for follow up. Patients’ permission to participate in the study was ensured by a consent form. It was translated and explained to the participants in their native language and signed by them. Consent was obtained by the parents and/or guardians for children below the age of 18.

Our inclusion criteria comprised of individuals with rheumatic diseases including the following types: Degenerative (osteoarthritis, cervical or lumbar spondylosis), inflammatory (rheumatoid arthritis and dermatomyositis), soft tissue rheumatism (non-specific low back pain, fibromyalgia, shoulder pain syndrome, carpal tunnel syndrome and tendinitis), crystal-induced (gout), autoimmune (systemic lupus erythematosus and scleroderma), metabolic (osteoporosis) and spondyloarthropathy (ankylosing spondylitis and psoriatic arthritis). Discrimination on the basis of gender, socioeconomic status, religion, and ethnicity was forgone. However, age ranges from 11 years to 90 years were approached for the interviews. The age groups were subdivided into four categories: adolescents from 11 years of age to 17 years of age, young adults from 18 years of age to 40 years of age, adults from 41 years of age to 65 years of age and older adults from 66 years of age to 90 years of age. Exclusion of traumatic injuries and bone tumors was implied.

The questionnaire was based on a total of 20 questions directed towards the diagnosed cases of rheumatic diseases, mainly focusing on their symptoms, duration of illness, presence of comorbidities, genetic background and the therapy they are undergoing along with compliance. The interviews were conducted by research participants, who were guided to wear identical lab coats and explained the questions to each interviewee in English and Urdu.

The sample size was calculated through Open Epi version 3.01 considering prevalence of rheumatic diseases to be 15.1%; a confidence level of 99% was taken with a confidence limit of 5% [10]. The computed sample size was 341, however, keeping our margin we interviewed a total of 346 people using non-probability convenience sampling. Bias was reduced by following the American Rheumatological Association Diagnostic criteria. The data was entered and analyzed using Statistical Package for the Social Sciences (SPSS) version 23.0 (IBM Corp., Armonk, New York). Frequency and percentages were calculated for categorical responses. Simple statistical analysis of frequency was done, whereas, chi-square test was employed to study associations with gender, age groups, and comorbidities. A p-value of less than 0.05 was considered significant.

## Results

During the study period, a total of 2000 patients visited the orthopedic OPD, 346 of which were diagnosed cases of rheumatic diseases, yielding a prevalence of 17.3%. The mean age of rheumatic patients who partook in the study was 46.15 ± 15.49 (Range: 12 – 84). Females were notably predominant in all diseases with the exception of ankylosing spondylitis, which had an equal male to female ratio. Osteoarthritis was the most common rheumatic disease, followed by non-specific low back pain and rheumatoid arthritis. Commonly encountered patients were adults, followed by a near equal frequency of young adults while few cases of older adults and even fewer cases of adolescents were seen. Diabetes and hypertension were observed as singleton comorbidities in 23 (6.6%) and 101 (29.2%) patients respectively, conjointly in 41 (11.9%) participants while being non-existent in 181 (52.3%) subjects. The prevalence, frequency, percentages, and distribution of rheumatic diseases amongst males and females are listed in Table 1 while Table 2 shows the distribution of frequency and percentages of various age groups.

**Table 1:** Prevalence and gender distribution of rheumatic diseases

Disease	Prevalence	Frequency	Percentage	Female (%)	Male (%)
Osteoarthritis	7.35%	147	42.5	126 (85.71)	21 (14.28)
Non-specific low back pain	3.95%	79	22.8	76 (96.2)	3 (3.8)
Rheumatoid arthritis	2.55%	51	14.7	49 (96.08)	2 (3.92)
Cervical or lumbar spondylosis	0.7%	14	4.0	11 (78.57)	3 (21.42)
Osteoporosis	0.7%	14	4.0	13 (92.85)	1 (7.14)
Shoulder pain syndrome	0.45%	9	2.6	6 (66.66)	3 (33.33)
Systemic lupus erythematosus	0.45%	9	2.6	6 (66.66)	3 (33.33)
Gouty arthritis	0.4%	8	2.3	7 (87.5)	1 (12.5)
Tendinitis	0.25%	5	1.4	5 (100)	0
Ankylosing spondylitis	0.1%	2	0.6	1 (50)	1 (50)
Carpal tunnel syndrome	0.1%	+2	0.6	2 (100)	0
Vasculitis	0.1%	2	0.6	2 (100)	0
Dermatomyositis	0.05%	1	0.3	1 (100)	0
Fibromyalgia	0.05%	1	0.3	1 (100)	0
Psoriatic arthritis	0.05%	1	0.3	1 (100)	0
Scleroderma	0.05%	1	0.3	1 (100)	0

**Table 2:** Frequency and percentages of rheumatic diseases in various age groups

Age Groups	Frequency	Percentage
Adolescents (11-17)	7	2
Young Adults (18-40)	141	40.8
Adults (41-65)	158	45.7
Older adults (66-90)	37	10.7

Males with diagnosed rheumatic diseases were 38 (11%) in number, their blights contributing to one-sixth cases of osteoarthritis, one-fourth of cases of cervical or lumbar spondylosis, one-third of cases of shoulder pain syndrome and systemic lupus erthematosus each, and a single case of osteoporosis and gouty arthritis each. In contrast to females, close to none cases of males were seen in non-specific low back pain and rheumatoid arthritis. Tendinitis, carpal tunnel syndrome, vasculitis, dermatomyositis, fibromyalgia, psoriatic arthritis, and scleroderma were not chanced upon in males, considering that each of these had very low prevalence. Non-specific low back pain, systemic lupus erthematosus, and shoulder pain syndrome were the only diseases to have a significant association with gender, their p-values being 0.02, 0.03 and 0.03 respectively.

Osteoarthritis was significantly seen in young adults, adults and older adults (p<0.05); thirty-five (23.8%) of the osteoarthritis afflicted patients significantly suffered from diabetes (p-value = 0.029) while 66 (44.9%) were hypertensive (p-value = 0.21). Non-specific low back pain was significantly seen in young adults and adults (p<0.05), however, only a few cases were identified in adolescents. As per data, eight (10.1%) were diabetic, bearing a significant association (p-value = 0.029) while 31 (39.2%) had hypertension (p-value = 0.711). Rheumatoid arthritis was seen mainly in young adults and adults having a frequency of 26 (51%) and 20 (39.2%) respectively, while three (5.9%) cases were seen in adolescents. Six (11.8%) patients suffered from diabetes (p-value = 0.18) while 20 (39.2%) were hypertensive (p-value = 0.774). Tables 3-4 show the frequency and p-values of osteoarthritis and non-specific low back pain within different age groups.

**Table 3:** Distribution of osteoarthritis in different age groups

Age Groups	Frequency	P-value
18-40	32	0.001
41-65	88	0.001
66-90	26	0.001
Missing	1	-
Total	147	

**Table 4:** Distribution of non-specific low back pain in different age groups

Age Groups	Frequency	P-value
11-17	2	0.849
18-40	55	0.001
41-65	17	0.001
66-90	4	0.17
Missing	1	
Total	79	

The adolescent population was afflicted with only a handful of conditions, namely non-specific low back pain, rheumatoid arthritis and systemic lupus erthematosus, of which only the lattermost was statistically significant (p-value = 0.001). Cervical or lumbar spondylosis, gouty arthritis, osteoporosis, tendinitis, and vasculitis were mostly seen in adults. Shoulder pain syndrome was mostly found in young adults (p-value = 0.273) and was significantly affiliated with diabetes (p-value = 0.042) while systemic lupus erthematosus was the only disease to have a significant association with hypertension (p value = 0.011). Psoriatic arthritis stumbled upon in a single female, co-existed with diabetes significantly (p-value = 0.036) and hypertension (p-value = 0.230). Overall, diabetes and hypertension whether individually or in conjunction, were found in 12 patients of osteoporosis, seven patients of cervical or lumbar spondylosis, six patients of shoulder pain syndrome, two patients each of gouty arthritis and tendinitis, one patient each of systemic lupus erthematosus, carpal tunnel syndrome, ankylosing spondylitis, fibromyalgia, and psoriatic arthritis. Diseases which were devoid of both the comorbidities were dermatomyositis, scleroderma, and vasculitis.

## Discussion

Rheumatic diseases have been deemed as inherently disabling entities. In the year 2015, they were responsible for 16.4%–20.9% of the global years lived with disability (YLDs) [11]. As a consequence of this debilitating nature, the afflicted individuals are cursed with an impaired quality of life and subsequently, missed workdays. In developing countries like Pakistan, where more than half of the population is dependent on daily wages as an only source of income, missing even a day’s worth of work can be gravely consequential. Moreover, inaccessibility to an effective healthcare system and delayed diagnosis further add to this hardship; ergo, timely provision of an adequate healthcare is both a basic right and a fundamental need of the people in developing countries and can be made attainable with the help of relevant research. Thus, we set out to evaluate the prevalence of rheumatic diseases within our population and obtained a result of 17.3%. According to Oguntona et al, the prevalence of rheumatic diseases in a hospital community of Nigeria was 15.1% [10]. Whereas, according to the Centers for Disease Control and Prevention March 2017 Vital Signs, the prevalence was 22.7% in the United States [12]. The discrepancy in our statistics with the United States' can be justified by the higher rates of obesity and increased life expectancy in their population. Osteoarthritis was the predominant malady with a prevalence of 7.4%. According to a prior study, the prevalence of osteoarthritis in Canadians aged 20 and above was 37% [13]. However, the number dwindled to 1.79% in Nigeria [10]. This discordance can be attributed to the proportional relation between disease prevalence and obesity rates in the respective countries [14]. Next in line was non-specific low back pain with a prevalence of 3.9%. A prior research conducted by Clémence et al showed a prevalence of 12.5% in France [15]. The decreased prevalence in Pakistan may be a result of comparatively low levels of obesity, superior pain tolerance, laborious way of life and a deprivation of medical insurance [14,16]. Rheumatoid arthritis was the third most common rheumatic disease with a prevalence of 2.5%. The prevalence of rheumatoid arthritis in the Chippewa Indians of central Minnesota, USA was 6.8% [17]. Our data adheres to the findings in previous researches that suggest that rheumatoid arthritis has a higher rate of prevalence in high-income countries than in the low- and middle-income countries [18], urbanization being the most plausible factor.

Unexpectedly, osteoarthritis was found to be statistically significant with the young adult population. Obesity, genetic factors, congenital anomalies of joints, vocational, and leisure activities such as contact sports as well as recurrent joint trauma and endocrine disorders like diabetes are thought to be the predisposing agents [19]. In contrast to the studies conducted previously, our data suggests a female preponderance in gouty arthritis [10, 20]. One would expect all of these women to be post-menopausal as estrogen has a uricosuric effect and thus, is protective against gout in the reproductive years. However, three out of the afflicted seven females were pre-menopausal. We believe this to be a result of the hormonal fluctuations in response to consumption of unsound chicken available in our region.

Majority of the plight of non-specific low back pain was borne by females (p-value 0.02). This gender bias was in corroboration with the studies conducted previously [21-23]. Menstruation, increased sensitivity to pain, pregnancy, physical distress of upbringing progeny and abdominal weight gain around the time of menopause are all thought to be the likely factors [24-26]. Non-specific low back pain was found to be significantly associated with young adults and adults. This can be explained on account of their sedentary lifestyle, lack of physical activity, overwhelming amounts of work, excruciating mental stress and obesity [27]. Furthermore, this ailment along with shoulder pain syndrome was also significantly associated with diabetes mellitus. The altered collagen, as a result of interaction with advanced glycation end products (AGEs), is deposited in the connective tissue surrounding the joints and thus compromises the structural and mechanical integrity of these joints, eventually leading to musculoskeletal infirmities [28].

The data in this study may help in contributing to the development of such healthcare facilities that will effectively screen the incoming patients and allocate them as those plagued by a rheumatic disease or not. This can be accomplished by creating awareness amongst the general population regarding rheumatic diseases and its risk factors, educating the primary health care physicians and paramedical professionals, and conducting more epidemiologic and etiologic researches featuring rheumatic diseases in the country.

The inflow of patients was more than our team could cover and in spite of exerting our best efforts, this emerged as the predominant limitation. Secondly, our study was confined to a singular department in a tertiary care setup and thus cannot exhibit the accurate prevalence of rheumatic diseases in our population, which in all likelihood is expected to be much higher than the one procured in our study. Our research technique was non-probability convenience sampling and as it is highly prone to selection bias, this too surfaced as a major limitation. Discrepancies in translating English to Urdu led to a communication barrier. A few patients who could have been diagnosed with rheumatic diseases did not follow up with the lab and/or radiological investigations ordered, thus, were missed.

## Conclusions

To conclude, this study exhibited the prevalence of different rheumatic diseases and demonstrated its burden within different age groups and genders, hinting at various risk factors that should be explored further for the said ailment. This data could aid tertiary care hospitals for the provision of sound healthcare and arrangement of community based programs to spread awareness amongst the mass population of developing countries.
